# The Effect of Antidepressants on the Course of Inflammatory Bowel Disease

**DOI:** 10.1155/2018/2047242

**Published:** 2018-09-09

**Authors:** Barry J. Hall, P. John Hamlin, David J. Gracie, Alexander C. Ford

**Affiliations:** ^1^Leeds Gastroenterology Institute, St. James's University Hospital, Leeds, UK; ^2^Leeds Institute of Biomedical and Clinical Sciences, University of Leeds, Leeds, UK

## Abstract

**Background and Aims:**

Mood may have an important role in the natural history of inflammatory bowel disease (IBD). However, the impact of antidepressant use on prognosis is unknown. We aimed to address this in a longitudinal study in a referral population.

**Methods:**

We collected demographic data, clinical disease activity and mood using validated questionnaires, and antidepressant use at baseline. Longitudinal disease activity was defined by disease flare or need for glucocorticosteroids, escalation of medical therapy, hospitalisation, or intestinal resection. We compared rates of these over a minimum period of 2 years according to antidepressant use at baseline.

**Results:**

In total, 331 patients provided complete data, of whom 54 (15.8%) were taking an antidepressant at study entry. Older age, female gender, and abnormal mood scores were associated with antidepressant use. During longitudinal follow-up, there was a trend towards lower rates of any of the four endpoints of IBD activity of interest in patients with abnormal anxiety scores at baseline and who were receiving an antidepressant (42.3% versus 64.6%, P = 0.05). Based on univariate Cox regression analysis, there was a trend towards lower rates of escalation of medical therapy among patients receiving antidepressants at baseline (hazard ratio (HR) = 0.59; 95% confidence interval (CI) 0.35-1.00, P = 0.05). None of the differences observed persisted after multivariate Cox regression.

**Conclusions:**

Antidepressants may have some beneficial effects on the natural history of IBD, but larger studies with longer follow-up are required. Whether these effects are limited to patients with abnormal mood remains uncertain.

## 1. Introduction

Crohn's disease (CD) and ulcerative colitis (UC) are chronic inflammatory organic disorders of the gastrointestinal (GI) tract of unknown aetiology, which may arise from dysregulation of both the innate and adaptive immune systems, leading to persistent and damaging inflammation of the intestine. Conventional therapies for inflammatory bowel disease (IBD) include glucocorticosteroids, 5-aminosalicylates, immunosuppressants, and biological therapies [1–7], with the aim of treatment being to induce remission of GI inflammation and prevent future disease relapse.

Patients with IBD appear to be at increased risk of exhibiting mood disorders, such as anxiety or depression, compared with healthy individuals [8–12]. However, whether this is a consequence of, or an etiological factor in, GI inflammation remains uncertain. In people with functional GI disorders, such as irritable bowel syndrome, longitudinal studies suggest that there is an increased likelihood of asymptomatic people who demonstrate anxiety or depression at baseline developing GI symptoms* de novo *[13]. This raises the possibility that coexistent anxiety or depression in patients with IBD, particularly if unrecognised or untreated, may have a deleterious effect on the natural history of IBD.

Evidence to support a role of abnormal mood in subsequent IBD activity comes mainly from animal models. Studies have demonstrated that in mouse models of quiescent colitis, inducing depression leads to reactivation of GI inflammation [14] but that this can be prevented by preadministration of antidepressants [15]. There is also limited evidence in humans to suggest that acute psychological stress induces proinflammatory cytokine production in both the serum and the mucosa of IBD patients [16]. Studies that have examined the effect of psychological comorbidity on the natural history of IBD suggest that patients with coexistent mood disorders have higher rates of disease relapse, escalation of therapy, and IBD-related surgery than patients with IBD without psychological disorders [17–20]. Despite this, the benefit of treatments, such as psychological therapies or antidepressants, which target mood abnormalities in patients with IBD is uncertain. A systematic review and meta-analysis of 14 randomised controlled trials (RCTs) of cognitive behavioural therapy, hypnotherapy, and other psychological interventions failed to demonstrate any effect on subsequent disease activity [21]. However, none of the included trials recruited only patients with psychological comorbidity. Another systematic review examining the effect of antidepressants on the course of IBD identified 15 studies [22], but the majority of these were case series or case reports. There were few longitudinal studies and only one small pilot RCT of fluoxetine in CD [23], which appeared to be of no benefit in terms of maintenance of disease remission.

This is an important issue, as patients with quiescent IBD may be at a lower risk of subsequent relapse of disease activity if coexisting mood disorders are identified and treated. This could lead to a more benign disease course, with fewer episodes of flares of disease activity and reductions in treatment escalation, hospitalisation, or surgery. There is, therefore, a need for epidemiological studies that examine the effects of antidepressants on the course of IBD in a setting that is generalisable to the type of patients with IBD seen in secondary care. We have therefore conducted a longitudinal follow-up study examining these issues in a large number of patients with IBD. Our* a priori* hypothesis was that there would be lower rates of, and longer time of onset to, objective markers of subsequent disease activity among those patients prescribed antidepressants at baseline.

## 2. Materials and Methods

### 2.1. Participants and Setting

Individuals recruited into a previous cross-sectional survey were included in this longitudinal follow-up study [24, 25]. All patients had an established radiological, histological, or endoscopic diagnosis of CD or UC and were aged ≥16 years at the time of baseline recruitment. Exclusion criteria were an inability to understand written English, a diagnosis of IBD-unclassified, and anyone with an end ileostomy or colostomy, due to the difficulties in assessing disease activity indices in these patients. Participants were followed up after a minimum period of 2 years had elapsed from initial recruitment. The longitudinal follow-up study was approved by the local research ethics committee in September 2014 (REC ref: 12/YH/0443), and data collection continued until June 2017. Study findings were reported in accordance with the STROBE guidelines for observational studies [26].

### 2.2. Data Collection and Synthesis

Date of recruitment into the original cross-sectional survey, demographic data, type of IBD, medication use for both IBD and non-IBD-related disease, and data concerning anxiety, depression, somatisation, and quality of life data were recorded at baseline, as described in the original cross-sectional survey [24, 25].

#### 2.2.1. Definition of Disease Activity, Anxiety or Depression, and Somatisation

At baseline assessment, a combination of disease activity indices (the Harvey-Bradshaw index (HBI) for CD [27] and the simple clinical colitis activity index (SCCAI) for UC [28]) were utilised. Clinical remission was defined as a score <5 for both CD and UC, as has been recommended by previous investigators [29, 30].

Anxiety and depression data were collected using the hospital anxiety and depression scale (HADS) [31]. This 14-item questionnaire consists of seven questions screening for the presence of anxiety symptoms and seven for depression symptoms, with a 4-point response for each item, ranging from 0 to 3. The total HADS score ranges from a minimum of 0 to a maximum of 21 for both anxiety and depression. Severity was categorised, according to total HADS score, into normal (total HADS depression or anxiety score 0-7), borderline normal (8-10), and abnormal (≥11) as previously recommended [31].

Somatisation data were collected using the patient health questionnaire-15 (PHQ-15), which is derived from the validated full PHQ [32]. Individuals were asked to rate the severity of 15 predefined symptoms as “not bothered at all” (scored as 0), “bothered a little” (scored as 1), or “bothered a lot” (scored as 2). Therefore the total PHQ-15 score ranges from a minimum of 0 to a maximum of 30. Somatisation severity was categorised, using the total PHQ-15 score, into high (total PHQ-15 ≥15), medium (10-14), low (5-9), and minimal (≤4) levels of somatisation severity, again as previously recommended [32].

#### 2.2.2. Antidepressant Use

Use of antidepressants was captured at the baseline visit. We recorded the regular prescription of any of the following drugs at study entry: selective serotonin reuptake inhibitors (SSRIs) including fluoxetine, fluvoxamine, paroxetine, sertraline, citalopram, escitalopram; tricyclic antidepressants (TCAs) including amitriptyline, nortriptyline, imipramine, clomipramine, desipramine, doxepin, or dosulepin; serotonin and noradrenaline reuptake inhibitors (SNRIs) including venlafaxine and duloxetine; tetracyclic antidepressants including mirtazapine; and atypical antidepressants, such as trazodone.

#### 2.2.3. Longitudinal Objective Assessment of IBD Activity

Objective assessment of disease activity during longitudinal follow-up was made by detailed case note review by a sole investigator (DJG), blinded to the baseline questionnaire data. The case notes of each of the patients included at baseline were assessed for the following four clinical endpoints, with the date of each endpoint recorded, where applicable: documented glucocorticosteroid prescription or a flare of disease activity identified by physician's global assessment; escalation of medical therapy due to uncontrolled disease activity, as judged by a physician's global assessment; hospitalisation secondary to objectively confirmed IBD activity; and intestinal resection. All these decisions were made as per usual care, as this was a noninterventional study conducted in routine clinical practice. Escalation of medical therapy in response to therapeutic drug monitoring, but in the absence of inflammatory activity, was not included as an endpoint, nor was surgical intervention for isolated perianal Crohn's disease.

### 2.3. Statistical Analysis

Baseline demographic, disease-related, and psychological data for all participants were compared between those taking and not taking antidepressants at study entry. Due to multiple comparisons we considered a 2-tailed P value of <0.01 to be statistically significant for these analyses. Proportions of patients experiencing the occurrence of one, or any, of the four objective markers of IBD activity during longitudinal follow-up were then compared between those patients taking an antidepressant at baseline and those who were not. We planned to perform subgroups analyses according to type of antidepressant used (SSRI, TCA, SNRI, or tetracyclic) as well as presence or absence of anxiety and/or depression at baseline, to assess whether these had any impact on the association between antidepressant use and IBD activity during longitudinal follow-up. We compared proportions in all these analyses using a *χ*^2^ test for categorical variables and an independent samples* t*-test for continuous data.

We then performed univariate Cox regression analysis to examine the effect of antidepressant use on IBD activity during longitudinal follow-up for all patients, and according to presence or absence of anxiety and/or depression at baseline. Independent predictors of the occurrence of any of the objective disease activity outcomes of interest were then determined by performing multivariate Cox regression analysis to control for antidepressant use, as well as all other baseline data. The results of these analyses were expressed as hazard ratios (HR) with 95% confidence intervals (CI). As our hypothesis that there would be lower rates of objective markers of IBD activity among those patients prescribed antidepressants at baseline was defined* a priori*, for these analyses we considered a 2-tailed P value of <0.05 to be statistically significant. All statistical analyses were performed using SPSS for Windows version 22.0 (SPSS Inc., Chicago, IL, USA).

## 3. Results

In total, 54 (15.8%) of 342 patients were taking an antidepressant at study entry. Of these, 32 (59.3%) were taking an SSRI, 17 (31.5%) a TCA, two (3.7%) an SNRI, two (3.7%) both an SSRI and a TCA, and one (1.9%) trazodone. Older age and female gender were both associated with antidepressant use, and there was a trend towards a higher body mass index (BMI) among those using antidepressants (Table 1). Patients with abnormal HADS anxiety or depression scores and those with high somatisation levels were more likely to be taking an antidepressant at baseline (P < 0.001).

### 3.1. Effect of Antidepressant Use on Subsequent IBD Activity

Of these 342 patients, 331 (96.8%) provided complete follow-up data for longitudinal IBD activity after case note review and were included in subsequent analyses. In all, 142 (42.9%) of 331 patients had a flare of disease activity or required glucocorticosteroid therapy during the 2-year follow-up period. Furthermore, 137 (41.4%) patients underwent escalation of medical therapy in response to uncontrolled disease activity. In terms of hospitalisation and intestinal resection, 43 (13.0%) and 19 (0.6%) patients experienced these endpoints of interest, respectively. There were no differences in proportions of patients experiencing a flare of disease activity or glucocorticosteroid use, need for escalation of therapy, hospitalisation, intestinal resection, or any of these endpoints according to use of antidepressants at baseline (Table 2). Limiting our analyses for flare of disease activity or requiring glucocorticosteroid therapy or escalation of medical therapy to only those who were in clinical remission at baseline did not affect our findings—9 (39.1%) of 23 patients receiving antidepressants experienced a flare of disease activity or needed a prescription for glucocorticosteroids versus 49 (32.0%) of 153 not receiving (P = 0.50); 7 (30.4%) of 23 patients receiving antidepressants required escalation of therapy versus 60 (39.2%) of 153 nor receiving (P = 0.42).

**Figure 1:**
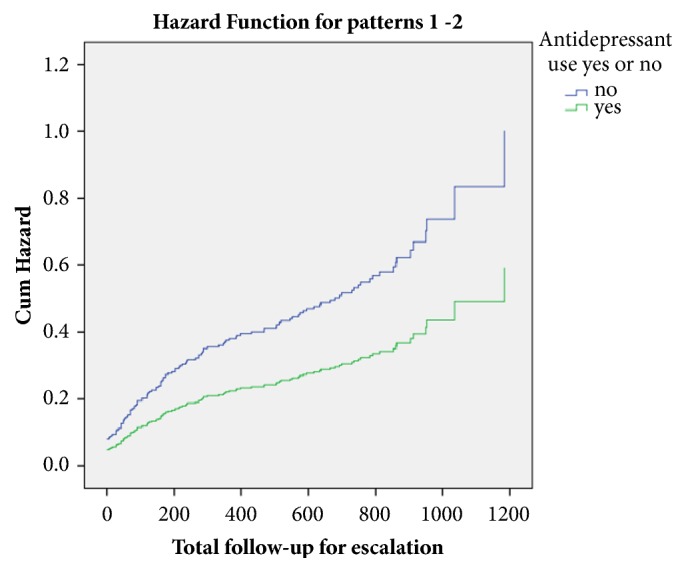
Cumulative hazard for escalation of medical therapy in response to uncontrolled IBD between patients receiving or not receiving antidepressants.

Based on univariate Cox regression analysis, there was a trend towards lower rates of escalation of medical therapy among patients receiving antidepressants at baseline, compared with those who were not (HR = 0.59; 95% CI 0.35 to 1.00, P = 0.05) (Table 3, Figure 1), but this was no longer the case based on multivariate Cox regression controlling for all baseline data.

**Figure 2:**
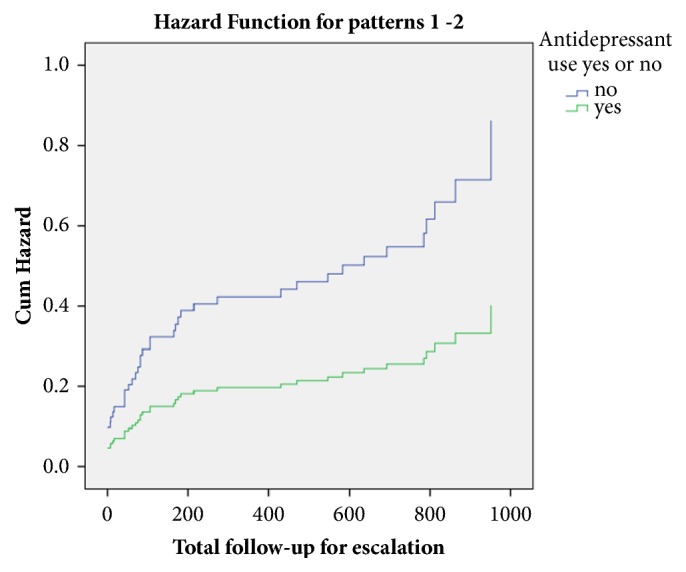
Cumulative hazard for escalation of medical therapy in response to uncontrolled IBD between patients with abnormal anxiety or depression scores at baseline receiving or not receiving antidepressants.

We performed subgroup analyses according to presence or absence of anxiety and/or depression at baseline (Table 2). There was a trend towards lower rates of escalation of therapy among those with either abnormal anxiety or depression scores at baseline and who were receiving an antidepressant, compared with those with either abnormal anxiety or depression scores at baseline but who were not receiving an antidepressant (25.5% versus 46.6%, P = 0.06). There were significantly lower rates of escalation of therapy among patients with abnormal anxiety scores at baseline and who were receiving an antidepressant, compared with those with abnormal anxiety scores at baseline but who were not receiving an antidepressant (23.1% versus 47.7%, P = 0.03). Finally there was a trend towards lower rates of any of the four endpoints of IBD activity of interest in patients with abnormal anxiety scores at baseline and who were receiving an antidepressant, compared with those with abnormal anxiety scores at baseline but who were not receiving an antidepressant (42.3% versus 64.6%, P = 0.05). Based on univariate Cox regression analysis, again there was a trend towards lower rates of escalation of medical therapy among patients with either abnormal anxiety or depression scores at baseline who were receiving antidepressants, compared with those with either abnormal anxiety or depression scores at baseline but who were not (HR = 0.47; 95% CI 0.21 to 1.05, P = 0.07) (Table 3) (Figure 2). However, this was no longer the case based on multivariate Cox regression.

### 3.2. Effect of SSRI Use on Subsequent IBD Activity

In total, 33 (61.1%) of all 54 patients taking an antidepressant at baseline were receiving an SSRI. There were no significant differences in the proportions of patients prescribed an SSRI experiencing one, or any, of the endpoints of interest, compared with those who were not (Table 4).

**Figure 3:**
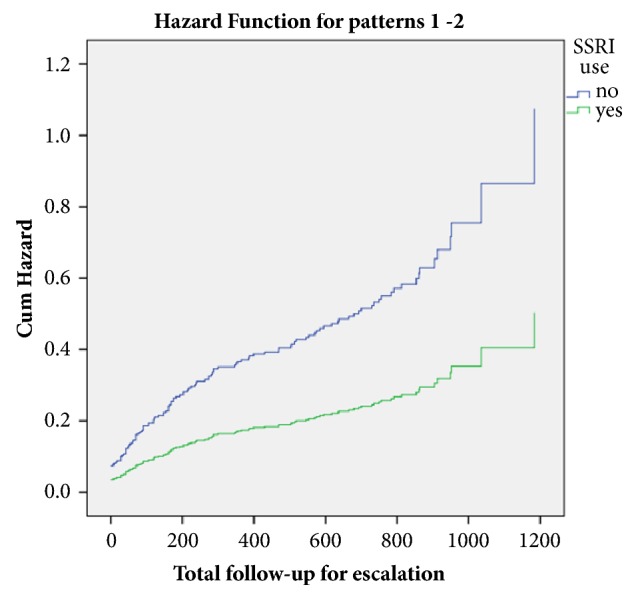
Cumulative hazard for escalation of medical therapy in response to uncontrolled IBD between patients receiving or not receiving SSRIs.

Based on univariate Cox regression analysis, there was a significantly lower rate of escalation of medical therapy among patients receiving SSRIs at baseline, compared with those who were not (HR = 0.47; 95% CI 0.24 to 0.93, P = 0.03) (Table 3) (Figure 3). This was no longer observed based on multivariate Cox regression.

Due to a reduced number of individuals taking SSRIs we were unable to perform subgroup analyses according to the presence of only abnormal anxiety scores, or only abnormal depression scores, at baseline. In the remaining analyses, there were trends towards higher rates of intestinal resection among patients with normal anxiety and depression scores at baseline who were taking SSRIs (15.4% versus 3.9%, P = 0.06) and lower rates of hospitalisation among patients with either abnormal anxiety or depression scores at baseline receiving SSRIs (0% versus 16.4%, P = 0.05) (Table 4). Based on both univariate (Table 3) and multivariate Cox regression analysis, there were no significant differences in rates of flare or glucocorticoid prescription, escalation of medical therapy, hospitalisation, or intestinal resection among SSRI users according to presence or absence of abnormal anxiety or depression scores at baseline.

## 4. Discussion

Results from univariate Cox regression analysis in this longitudinal study, adjusted for total duration of follow-up among all individuals, suggest a trend towards lower rates of subsequent escalation of medical therapy among patients with IBD receiving antidepressants at baseline, and this persisted when only those patients with abnormal anxiety or depression scores at study entry were considered in the analysis. Similar findings were observed when only those using SSRIs at baseline were considered in the analysis. However, none of these associations remained statistically significant based on multivariate Cox regression analysis. Our findings, although interesting, should be considered as preliminary only, and larger studies with longer follow-up, or rigorous RCTs, which are powered to examine this issue specifically, will be required before the true effect of antidepressant use on objective disease endpoints in patients with IBD is known.

We obtained longitudinal disease activity outcome data for a cohort of 331 patients with IBD. The observational design and the use of a secondary care population mean that our findings are likely to be generalisable to the majority of patients with IBD seen outside specialist centres. Our use of validated questionnaires to assess for the presence of anxiety [31], depression [31], or somatisation [32] at baseline is a further strength. We controlled for differences in duration of follow-up between those receiving, or not receiving, antidepressants at baseline and all other baseline data, using multivariate Cox regression. Finally, objective longitudinal disease activity assessment data were collected blinded to baseline disease activity and psychological data, therefore eliminating researcher confirmation bias.

Weaknesses include the sample size and length of follow-up. Based on multivariate Cox regression analysis, there were no statistically significant differences observed in rates of any of the objective markers of disease activity according to antidepressant use at baseline, despite the fact that the absolute proportion of patients reaching these endpoints were generally lower among those taking antidepressants at baseline, particularly among those with abnormal anxiety and/or depression scores at baseline. This suggests that if our sample size had been larger, or the duration of follow-up longer, some of these comparisons may have become statistically significant. Alternatively, there may be a true beneficial effect that was lost when other factors were controlled for in our multivariate analysis. This could be due to either confounding or a loss of time contributed to the analysis from individuals with missing data for these demographic characteristics. Our assessment of psychological well-being was based on questionnaire responses at a single point in time, and our use of HADS scores as a marker of anxiety or depression could be criticised as, although these are widely used, their psychometric properties have been challenged [33]. Although we collected disease activity endpoints based on objectively defined criteria, there is the possibility that some of these endpoints, such as escalation of therapy, were reached based on symptom reporting alone, rather than true inflammatory activity. Due to the low event rate for some of the longitudinal disease activity outcomes, we chose not to present data on IBD patients dichotomised into those with UC and CD, or according to the use of antidepressant subclasses, other than SSRIs. Finally, use of antidepressants at baseline was classified according to a documented current prescription for the drugs of interest, but whether patients were actually adherent to their antidepressant therapy is uncertain. In addition, we did not record the discontinuation or commencement of antidepressant drugs during follow-up and therefore cannot exclude some misclassification of patients.

Due to the chronicity of their disease and the required shifts in coping and self-management skills over time, it is not surprising that patients with IBD are at increased risk of mental health disorders, yet previous studies have demonstrated that these coexistent psychological problems are often undertreated [34]. Undertreatment of mental health disorders can lead to long-term disability and a stigma against seeking treatment [35, 36]. Most IBD specialists have little formal training in the detection and management of such psychological comorbidities. This lack of training, combined with the potential hesitancy of many patients to report psychological symptoms, may influence therapy options. Although our study demonstrated that patients with abnormal anxiety or depression scores and those with high somatisation levels were more likely to be taking an antidepressant at baseline, the prevalence of abnormal anxiety scores, abnormal depression scores, and high somatisation scores in patients not receiving antidepressant therapy was also relatively high at 24.2%, 7.7%, and 20.7%, respectively.

Decreasing potential long-term sequelae by minimising the inflammatory burden in IBD patients is an important treatment strategy. There is some evidence, from studies in both animals and humans with IBD, to suggest that psychological comorbidity can trigger inflammatory activity [14, 16]. Furthermore, patients with IBD are at increased risk of psychological comorbidity, particularly depression and anxiety [37]. Thus, pharmacological therapy for mood disorders may have the potential to alter the natural history of CD and UC. Limited data, to date, have suggested improvements in mood, inflammatory activity, and quality of life with antidepressant therapy [23, 38]. Our own longitudinal follow-up data did not show any statistically significant differences in the proportion of patients experiencing endpoints consistent with inflammatory activity, according to use of antidepressants at baseline. However, the absolute proportions reaching these endpoints were generally lower among those prescribed antidepressants, particularly when only those with abnormal anxiety or depression scores were considered in the analysis.

Mechanisms of any beneficial effect of antidepressant drugs on the course of IBD are uncertain. Some antidepressants, like amitriptyline, appear to have both pain modifying properties [39] and direct effects on proinflammatory cytokines that may arise via actions on the nuclear factor-*κ*B and nitric oxide pathways, which are implicated in the pathogenesis of IBD [40]. The former may explain the trend towards a reduction in escalation of therapy we observed, although given that this was also observed when only patients taking SSRIs, which have less in the way of analgesic effects [39], were considered, this seems unlikely. Therefore, antidepressant therapy may indeed have a beneficial effect on inflammatory activity. However, differentiating the true inflammatory burden in a symptomatic patient with IBD and coexistent mood disorder can be difficult, as the available symptom scoring systems cannot confirm the true origin of symptoms, and patients with IBD with psychological comorbidity may be more likely to report GI symptoms in general [24, 25]. As a result patients with mood disorders and who were on antidepressants in our study may potentially have been less likely to be offered, or indeed accept, a glucocorticosteroid prescription, a step-up in therapy, hospitalisation, or surgery.

In summary, our longitudinal follow-up study demonstrated lower rates of some objective markers of disease activity among patients with IBD receiving antidepressants and SSRIs, at baseline, particularly among those with abnormal anxiety or depression scores at baseline. However, none were statistically significant based on multivariate Cox regression. Further observational studies or RCTs with longer follow-up, which are powered to examine this issue specifically, will be required before the true effect of antidepressant use on disease activity in patients with CD and UC is known.

## Figures and Tables

**Table 1:** Baseline demographic, disease-related, and psychological characteristics of patients with IBD according to use of antidepressants.

	**Prescribed Antidepressants at Baseline (n = 54) **	**Not prescribed Antidepressants at Baseline (n = 288) **	**P value** **∗**
**Mean age in years (SD) **	47.1 (13.8)	39.6 (16.2)	0.001

**Mean duration of follow-up in days (SD) for each endpoint**			
Glucocorticosteroid prescription or flare of disease activity	648.3 (409)	502.9 (361.8)	0.02
Escalation of medical therapy in response to uncontrolled disease activity	664.0 (399.1)	531.0 (345.3)	0.03
Hospitalisation due to disease activity (%)	841.4 (284.1)	733.2 (252.1)	0.01
Intestinal resection (%)	861.4 (265.6)	770.7 (218.1)	0.02

**Female gender (**%**) **	47 (87.0)	158 (54.9)	<0.001

**Caucasian ethnicity (**%**) **	51 (96.2)	267 (93.7)	0.47

**Married or co-habiting (**%**) **	27 (50.9)	165 (58.3)	0.32

**University/postgraduate (**%**) **	16 (29.6)	102 (36.0)	0.37

**Mean BMI (SD) **	28.0 (6.2)	25.6 (5.3)	0.01

**Tobacco user (**%**) **	14 (25.9)	48 (16.8)	0.11

**Alcohol user (**%**) **	31 (57.4)	200 (69.9)	0.07

**Crohn's disease (**%**) **	20 (37.0)	118 (41.0)	0.59

**5-ASA use (**%**) **	27 (50.0)	124 (43.1)	0.35

**Immunosuppressant use (**%**) **	16 (29.6)	117 (40.6)	0.13

**Biologic use (**%**) **	6 (11.1)	63 (21.9)	0.07

**Glucocorticosteroid use (**%**) **	5 (9.3)	26 (9.0)	0.96

**HBI/SCCAI <5 (**%**) **	23 (45.1)	159 (58.7)	0.07

**HADS anxiety scores at baseline (**%**)**			
Normal	16 (29.6)	167 (58.6)	
Borderline abnormal	10 (18.5)	10 (18.5)	
Abnormal	28 (51.9)	69 (24.2)	<0.001

**HADS depression scores at baseline (**%**)**			
Normal	29 (53.7)	230 (80.1)	
Borderline abnormal	11 (20.4)	35 (12.2)	
Abnormal	14 (25.9)	22 (7.7)	<0.001

**PHQ-15 somatisation scores at baseline (**%**)**			
Mild	0 (0)	50 (18.8)	
Low	8 (15.7)	83 (31.2)	
Medium	20 (39.2)	78 (29.3)	
High	23 (45.1)	55 (20.7)	<0.001

*∗*Independent samples t-test for continuous data and *χ*2 for comparison of categorical data.

**Table 2:** Effect of antidepressant use on subsequent development of IBD activity and according to anxiety and depression scores at baseline.

	**Antidepressant ** **Use:** **All Patients**			**Antidepressant ** **Use:** **Normal Anxiety and Depression Scores at Baseline**			**Antidepressant** **Use: Abnormal Anxiety or Depression Scores at Baseline**			**Antidepressant** **Use: Abnormal Anxiety Scores at Baseline**			**Antidepressant** **Use: Abnormal Depression Scores at Baseline**		
	**No**	**Yes**	**P value**	**No**	**Yes**	**P value**	**No**	**Yes**	**P value**	**No**	**Yes**	**P value**	**No**	**Yes**	**P value**
**Glucocorticosteroid prescription or flare of disease activity (**%**) **	121/279 (43.4)	21/52 (40.4)	0.69	82/203 (40.4)	10/25 (40.0)	0.97	38/73 (52.1)	11/27 (40.1)	0.32	35/65 (53.8)	10/26 (38.5)	0.19	9/21 (42.9)	6/14 (42.9)	1.00

**Escalation of medical therapy in response to uncontrolled disease activity (**%**) **	120/279 (43.0)	17/52 (32.7)	0.17	85/203 (41.9)	10/25 (40.0)	0.86	34/73 (46.6)	7/27 (25.9)	0.06	31/65 (47.7)	6/26 (23.1)	0.03	8/21 (38.1)	5/14 (35.7)	0.89

**Hospitalisation due to disease activity (**%**) **	38/279 (13.6)	5/52 (9.6)	0.43	26/203 (12.8)	4/25 (16.0)	0.66	12/73 (16.4)	1/27 (3.7)	0.09	11/65 (16.9)	1/26 (3.8)	0.10	2/21 (9.5)	0/14 (0)	0.23

**Intestinal resection (**%**) **	15/279 (5.4)	4/52 (7.7)	0.51	8/203 (3.9)	3/25 (12.0)	0.08	7/73 (9.6)	1/27 (3.7)	0.34	6/65 (9.2)	1/26 (3.8)	0.38	2/21 (9.5)	0/14 (0)	0.23

**Any adverse outcome (**%**) **	148/279 (53.0)	24/52 (46.2)	0.36	102/203 (50.2)	12/25 (48.0)	0.83	45/73 (61.6)	12/27 (44.4)	0.12	42/65 (64.6)	11/26 (42.3)	0.05	10/21 (47.6)	6/14 (42.9)	0.78

**Table 3:** Effect of antidepressant or SSRI use on subsequent IBD activity based on univariate cox regression analysis.

	**Glucocorticosteroid ** **prescription or flare of ** **disease activity**		**Escalation of Medical ** **Therapy in Response ** **to Uncontrolled ** **Disease Activity**		**Hospitalisation due to ** **Disease Activity**		**Intestinal resection**	
	**Hazard ratio** **(95**%** CI)**	**P value**	**Hazard ratio** **(95**%** CI)**	**P value**	**Hazard ratio** **(95**%** CI)**	**P value**	**Hazard ratio** **(95**%** CI)**	**P value**
**Antidepressant use:** **all patients **	0.76 (0.47-1.22)	0.25	0.59 (0.35-1.00)	0.05	0.59 (0.231.50)	0.26	1.15 (0.373.53)	0.81

**Antidepressant use: normal anxiety and depression scores at baseline **	0.77 (0.38-1.54)	0.77	0.72 (0.36-1.44)	0.35	1.01 (0.352.93)	0.98	2.15 (0.558.50)	0.27

**Antidepressant use: abnormal anxiety or depression scores at baseline **	0.63 (0.32-1.23)	0.17	0.47 (0.21-1.05)	0.07	0.20 (0.031.57)	0.13	0.37 (0.053.04)	0.36

**SSRI use: all patients **	0.74 (0.41-1.31)	0.30	0.47 (0.24-0.93)	0.03	0.35 (0.081.46)	0.15	0.86 (0.193.85)	0.84

**SSRI use: normal anxiety and depression scores at baseline **	0.76 (0.31-1.91)	0.56	0.49 (0.18-1.37)	0.18	0.87 (0.203.75)	0.86	2.39 (0.4712.2)	0.29

**SSRI use: abnormal anxiety or depression scores at baseline **	0.58 (0.27-1.26)	0.17	0.43 (0.17-1.11)	0.08	0.03 (0.0010.1)	0.24	0.03 (0.0073.9)	0.39

**Table 4:** Effect of SSRI use on subsequent development of IBD activity and according to anxiety and depression scores at baseline.

	**SSRI Use: All ** **Patients**			**SSRI Use: Normal ** **Anxiety and ** **Depression ** **Scores at Baseline**			**SSRI use: ** **Abnormal ** **Anxiety or ** **Depression ** **Scores at Baseline**		
	**No**	**Yes**	**P Value**	**No**	**Yes**	**P value**	**No**	**Yes**	**P value**
**Glucocorticosteroid prescription or flare of disease activity (**%**) **	121/279 (43.4)	14/33 (42.4)	0.92	82/203 (40.4)	6/13 (46.2)	0.68	38/73 (52.1)	8/20 (40.0)	0.34

**Escalation of medical therapy in response to uncontrolled disease activity (**%**) **	120/279 (43.0)	10/33 (30.3)	0.16	85/203 (41.9)	5/13 (38.5)	0.81	34/73 (46.6)	5/20 (25.0)	0.08

**Hospitalisation due to disease activity (**%**) **	38/279 (13.6)	2/33 (6.1)	0.22	26/203 (12.8)	2/13 (15.4)	0.79	12/73 (16.4)	0/20 (0)	0.05

**Intestinal resection (**%**) **	15/279 (5.4)	2/33 (6.1)	0.87	8/203 (3.9)	2/13 (15.4)	0.06	7/73 (9.6)	0/20 (0)	0.15

**Any adverse outcome (**%**) **	148/279 (53.0)	14/33 (42.4)	0.25	102/203 (50.2)	6/13 (46.2)	0.78	45/73 (61.6)	8/20 (40.0)	0.08

## Data Availability

The data used to support the findings of this study are available from the corresponding author upon request.

## Abbreviations

BMI: Body mass index
CD: Crohn's disease
CI: Confidence interval
GI: Gastrointestinal
HADS: Hospital anxiety and depression scale
HBI: Harvey-Bradshaw index
HR: Hazard ratio
IBD: Inflammatory bowel disease
PHQ-15: Patient health questionnaire-15
RCT: Randomised controlled trial
SCCAI: Simple clinical colitis activity index
SNRI: Serotonin and noradrenaline reuptake inhibitor
SSRI: Selective serotonin reuptake inhibitor
TCA: Tricyclic antidepressant
UC: Ulcerative colitis.
